# Circadian‐Inspired Hydrogel Moisture Electricity Generation

**DOI:** 10.1002/advs.77689

**Published:** 2026-09-09

**Authors:** Ziqi Li, Dilalanmu Ahemaitijiang, Hongfei Yang, Xuan Zhao, Mengjiao Pan, Jun Wan, Pingan Zhu, Dahua Shou

**Affiliations:** ^1^ Research Centre of Texitles for Future Fashion The Hong Kong Polytechnic University Kowloon Hong Kong; ^2^ Future Intelligent Wear Centre, PolyU‐Xingguo Technology and Innovation Research Institute, School of Fashion and Textiles The Hong Kong Polytechnic University Kowloon Hong Kong; ^3^ Research Institute for Intelligent Wearable Systems The Hong Kong Polytechnic University Kowloon Hong Kong; ^4^ Department of Mechanical Engineering City University of Hong Kong Kowloon Hong Kong

**Keywords:** hydrogel, moisture electricity generator, reversible electricity output

## Abstract

Energy harvesting from ambient moisture offers a ubiquitous solution to the energy crisis, free from geographical restrictions. While hydrogel‐based moisture electric generators (MEGs) show promise as scalable sustainable energy sources, they currently face significant challenges regarding performance degradation due to inevitable water saturation or desiccation. Here, we present a circadian‐inspired hydrogel MEG that exhibits sustained electricity generation by leveraging diurnal temperature‐moisture fluctuations—traditionally considered detrimental noise—as a critical driving force. By combining an asymmetric hygroscopic hydrogel architecture with a conditioning treatment, the device undergoes reversible internal water redistribution, which is closely associated with periodic reversal of the effective driving force for electrical output. The resulting MEG delivers a short‐circuit current density of 75 µA cm^−2^, an open‐circuit voltage of 0.4–1 V, and a power density of 16 µW cm^−2^, consistently maintaining performance over a 57‐day period. Electrical characterization, spectroscopy, and gravimetric analysis support a water‐redistribution mechanism for the observed reversible electrical current. Similar behavior was also observed in several related hydrogel system. This work highlights a previously underexplored operational mode in hydrogel moisture electricity generation and offers a promising design concept for sustained ambient energy harvesting under coupled thermal–moisture fluctuations.

## Graphical abstract

A circadian‐inspired hydrogel moisture‐electric generator enables sustainable electricity generation by autonomously current direction switching between daytime and nighttime. This reversible output is accompanied by inversion of the internal water gradient, which effectively reduce hydrogel fatigue and offers a new strategy for harvesting diurnal thermal fluctuations through dynamic water redistribution in hydrogel materials.

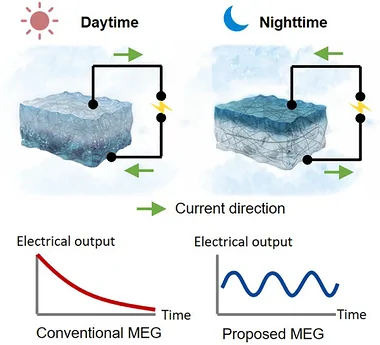

## 1 Introduction

Transitioning to sustainable energy sources is imperative to reduce reliance on finite fossil fuels and overcome geographical constraints inherent to centralized power generation [1, 2]. As a cornerstone of renewable energy, hydropower accounts for approximately half of global renewable electricity generation [3, 4, 5]. Generally, hydro‐energy harvesting strategies exploit bulk water [6, 7], water droplets [8], evaporation [9, 10], or atmospheric moisture [11, 12, 13]. However, the first three approaches each have inherent constraints: bulk water and droplets require specific kinetic inputs (e.g., waves or precipitation), while evaporation‑induced generation relies on a continuous water reservoir and its performance typically declines or ceases when the source is exhausted. In contrast, moisture electricity generation (MEG) captures energy directly from atmospheric moisture, a ubiquitous reservoir continuously replenished by the global water cycle and human activities. This distinctive feature positions MEG as a truly autonomous power solution, in principle independent of specific weather conditions or liquid water availability [13, 14, 15].

MEGs typically generate electricity by establishing a chemical or moisture gradient within host materials, driving mobile ions or charges from high to low concentration to produce a current [16]. Extensive efforts have been devoted to constructing such gradients using diverse materials, including carbon‐based nanomaterials (graphene [17], graphene oxides [17, 18]), conductive polymers [19, 20], MXenes [21], metal–organic frameworks [22], protein nanowires [13]. polyelectrolytes [23] and hydrogels [12, 24, 25]. Among these, hydrogel‐based MEGs have emerged as particularly promising candidates due to their strong hygroscopicity and intrinsically clean and green nature. Despite these advances, achieving long‐term power generation remains a formidable bottleneck. The fundamental issue lies in the thermodynamic tendency of hydrogels to equilibrate with the environment: prolonged exposure inevitably drives them toward either saturation (in high humidity) or desiccation (in low humidity or at elevated temperature), over which internal water redistributes and ion gradients dissipate, ultimately causing a collapse of electricity output [14, 26, 27].

Current strategies to extend MEG lifespan have largely focused on enhancing intrinsic material stability, thereby passively resisting environmental equilibrium. For instance, incorporating cellulose nanofibers [28] or constructing supramolecular networks [29] can bolster structural integrity, while bilayer aero‐hydrogel designs [30] optimize internal moisture transport. However, these methods merely delay the inevitable decay of the moisture gradient. Even in devices claiming long‐term operation, the current output often plummets to a fraction of its initial value after extended periods [31, 32, 33]. Notably, most existing approaches often overlook a key environmental variable: the natural fluctuation of ambient conditions. Rather than viewing environmental volatility as a disturbance to be resisted, it can instead be harnessed as an active thermodynamic driver to dynamically regulate the hydrogel's hydration state.

In this work, we introduce a circadian‑inspired strategy that utilizes ambient diurnal temperature fluctuations to drive a self‑sustaining moisture cycle. Specifically, we develop a polyvinyl alcohol‐sodium alginate‐Nafion (PVA‐SA‐Nf) hydrogel that harnesses the natural diurnal temperature variations to establish a reversible water concentration gradient. Unlike static systems, this asymmetric structure undergoes a dynamic “breathing” process: maintaining lower water content on the exposed surface during warmer periods (desorption) and replenishing moisture during cooler periods (adsorption). This temperature‑driven mechanism avoids saturation and desiccation, enabling the MEG to sustain a reversible ionic current with negligible performance degradation over at least 57 days and potentially much longer. By converting diurnal temperature cycles from a destabilizing factor into a built‑in thermodynamic engine, this work provides a generalizable framework for designing hydrogel‑based MEGs capable of spontaneous, long‑term stable moisture‑powered electricity generation.

## 2 Results and Discussions

### 2.1 Design of Circadian‐Inspired MEG

**FIGURE 1:**
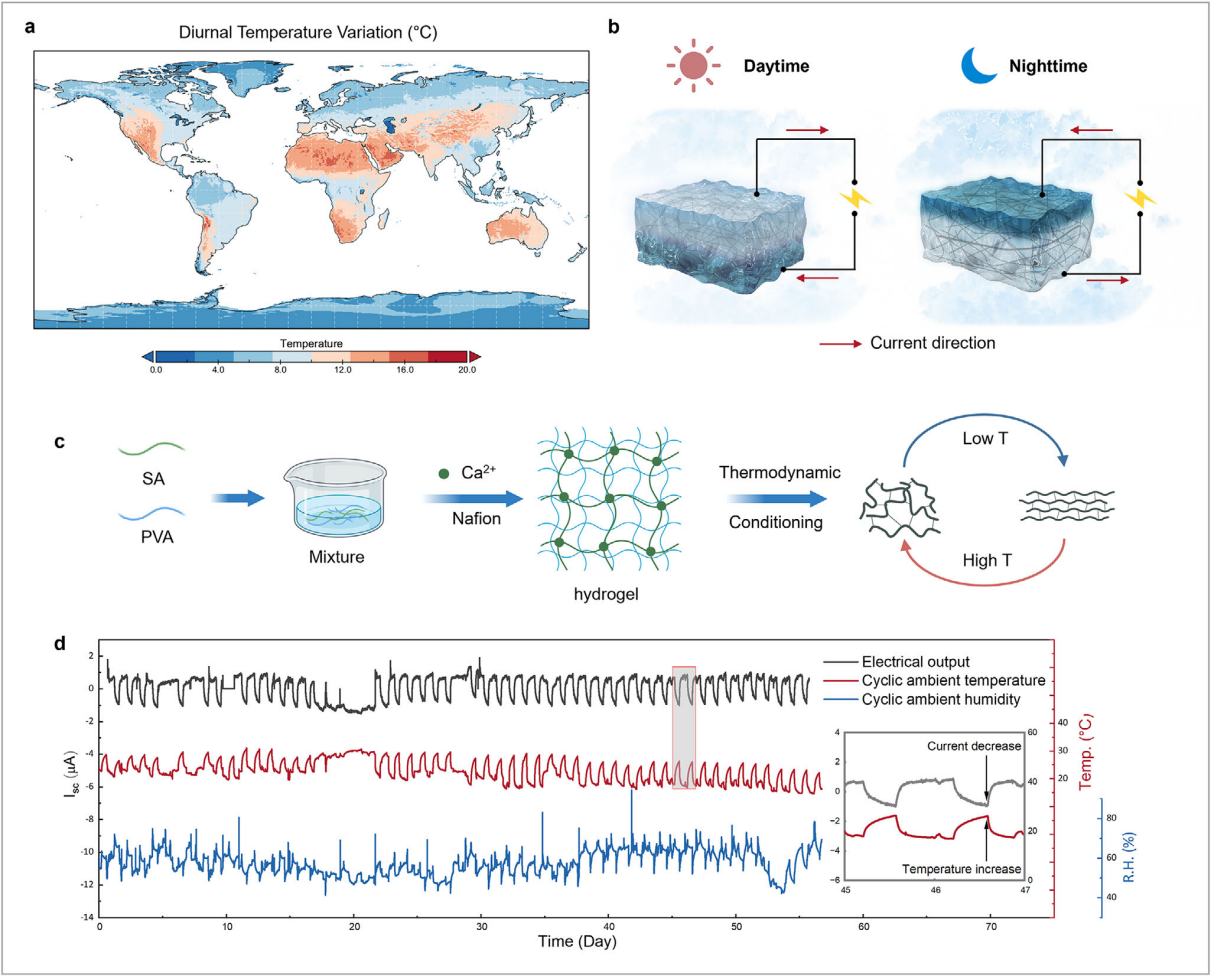
Circadian‐Inspired Hydrogel MEG. (a) Global map of the mean daily near‑surface (2 m) air‑temperature range (°C) for 1991–2020, highlighting the widespread availability of diurnal thermal fluctuations. Data were obtained from the Climate Data Store (CDS). (b) Schematic illustration of the PVA‑SA‑Nf hydrogel MEG showing reversible current directions under daytime and nighttime conditions. (c) Fabrication of PVA‐SA‐Nf hydrogel and subsequent thermodynamic conditioning treatment. (d) Long‐term continuous power generation performance of the hydrogel MEG recorded over 57 days under indoor diurnal conditions. The plot displays the real‐time monitoring of short‐circuit current (*I_SC_*), ambient temperature (Temp.), and relative humidity (RH). Inset: Magnified view demonstrating the synchronized response of the current output to the periodic ambient temperature fluctuations. Hydrogel dimension: 10 mm * 10 mm * 0.8 mm.

Harvesting energy spontaneously and effectively from the ambient environment is of paramount importance for the development of self‐powered systems. The atmosphere serves as a ubiquitous energy reservoir, continuously replenished by the global water cycle, where moisture conversion occurs across most habitable latitudes. (Figure S1) Coinciding with this moisture availability, widespread diurnal temperature variations—averaging 8°C–12°C in many regions—provide a powerful, yet often overlooked, thermodynamic driving force (Figure 1a). The coexistence of abundant atmospheric moisture and natural thermal cycling establishes the physical basis for our circadian‐inspired hydrogel MEG. Unlike conventional hydrogel‐based MEGs, which remain static and suffer from rapid decay during long‐term electric generation, our proposed MEG sustains a thermodynamic non‐equilibrium driven by diurnal thermal fluctuations, which is inspired by the circadian temperature rhythm. This dynamic adaptation results in a reversible current direction synchronized with diurnal temperature fluctuations, thereby realizing long‐term, sustainable generation (Figure 1b).

To harness these environmental dynamics, we engineered a PVA‐SA‐Nf hydrogel capable of “breathing” moisture in response to thermal fluctuations. Specifically, the hydrogel matrix was synthesized by blending PVA and SA, followed by the incorporation of CaCl_2_ and Nafion (Figure 1c). In this composition, PVA serves as the structural backbone, while SA enhances moisture uptake capability due to its abundant hydroxyl groups. Nafion, a polyelectrolyte, is introduced to facilitate proton transport, and CaCl_2_ further augments the hygroscopic capacity of the hydrogel. The resulting PVA‐SA‐Nf hydrogel was assembled into an asymmetric device configuration utilizing graphene and gold electrodes. A nanoporous polyethylene (PE) layer covers one side of the hydrogel, whereas the opposite side is directly exposed to air, creating asymmetric pathways for moisture transfer. The differential exposure of these interfaces (covered vs. exposed) establishes an initial water gradient across the hydrogel, driving water molecule diffusion and subsequent electricity generation. Instead of static synthesis, we employed a cyclic thermodynamic conditioning protocol to program the hydrogel's internal structure (Figures S2 and S3). This process induces a dynamic non‐equilibrium where polymer chains oscillate between extended (hydrated) and globule (dehydrated) states (Figure 1c), further enabling a reversible internal water gradient that drives directional ion transport. Control experiments including electrode symmetry, reversible electrodes, CaCl_2_ addition, PE coverage, and sealed‐device measurements support that the reversible electrical output is closely associated with asymmetric moisture transport and internal water redistribution in the hydrogel (Figures S4–S8).

Figure 1d demonstrates the exceptional long‐term sustainability of the MEG, recording continuous electrical output over 57 days under diurnal indoor conditions. Unlike conventional MEGs that suffer from rapid decay due to saturation or desiccation, our device maintained long‐term self‐sustaining output stability with negligible degradation (Figures S9 and S10 and Table S1). Most strikingly, the current direction exhibits a distinct diurnal reversal pattern, synchronizing perfectly with the circadian rhythm. As shown in the inset of Figure 1d, the current output displays an anti‐phase correlation with temperature: during daytime air‑conditioning, the temperature remains low, and the current is sustained, whereas at night, when the air‑conditioning is turned off and the temperature rises, the current reverses and varies accordingly. This phenomenon arises from a temperature‐driven reversal of the moisture gradient enabled by the asymmetric interface: at elevated temperatures, the air‐exposed surface dries more rapidly due to enhanced desorption, while the PE‐covered side retains water; at lower temperatures, the exposed surface more readily adsorbs moisture from ambient air and becomes wetter than the PE‐covered side, thereby inverting the internal gradient. This reversible “breathing” process effectively prevents the accumulation of water molecules (saturation) or excessive loss (desiccation), maintaining the hydrogel in a thermodynamically stable dynamic state. This temperature‑dominated mechanism highlights a distinct design and operating principle compared with previously reported humidity‑governed MEGs [24, 30], suggesting that ambient thermal fluctuations play a critical role in driving the internal water dynamics, enabling the MEG to sustain continuous power generation through a self‐regulated dynamic effect.

### 2.2 Electrical Output Performance

**FIGURE 2:**
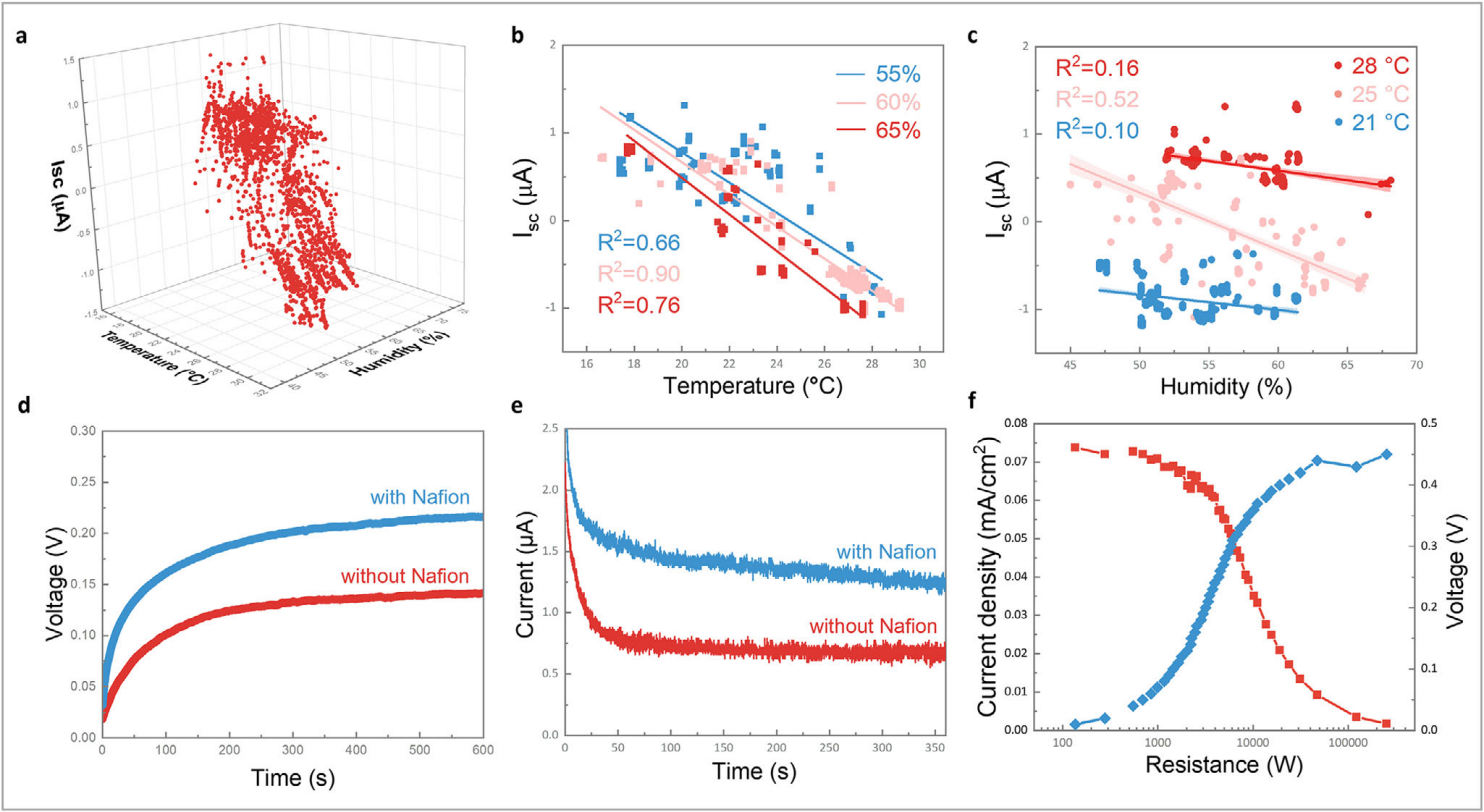
Electrical output performance of the PVA‐SA‐Nf MEG. (a) Three‐dimensional (3D) scatter plot mapping *I_SC_* as a function of humidity and temperature, revealing the influence of thermal conditions. (b) Linear correlation analysis between *I_SC_* and temperature at fixed RH of 55%, 60%, and 65%. (c) Linear correlation analysis between *I_SC_* and RH at fixed temperatures of 21°C, 25°C, and 28°C. (d) Open‐circuit voltage (*V_oc_*) and (e) *I_SC_* profiles comparing the MEGs fabricated with (blue) and without (red) Nafion, highlighting the role of the polyelectrolyte in enhancing output. (f) Current and voltage as a function of load resistance for the device which has a working area of 1 cm^2^, demonstrating a peak power output at ∼30 kΩ.

To elucidate the dependence of the electrical output on environmental factors, we constructed a three‐dimensional map of the short‐circuit current (*I_sc_*) against ambient temperature and relative humidity (Figure 2a). The profile reveals a complex interplay where the output is influenced by both parameters, yet with distinct sensitivities. Quantitative analysis using linear regression (Figure 2b) uncovers a strong negative correlation between *I_sc_* and temperature, with coefficients (R^2^) reaching 0.90, 0.76, and 0.66 at relative humidities of 60%, 65%, and 55%, respectively. This indicates a robust, temperature‑driven mechanism. In contrast, the correlation between *I_sc_* and humidity is significantly weaker (R^2^ values of 0.16, 0.52, and 0.10), suggesting that humidity fluctuations play a secondary role in this system (Figure 2c).

Detailed controlled experiments further confirm this temperature dependency. At constant humidity, the current magnitude exhibits a clear inverse relationship with temperature (Figures S11 and S12). Crucially, a polarity reversal point is observed around 25°C (Figure S13). This switch implies a fundamental inversion in the internal charge carrier dynamics, likely driven by the reversal of the water concentration gradient across the asymmetric hydrogel interface. Humidity response analysis confirms that the current direction is primarily dictated by the thermal state: positive at lower temperatures (e.g., 21°C, corresponding to adsorption) and negative at higher temperatures (e.g., 28°C, corresponding to desorption) (Figures S14 and S15). While humidity does influence the current magnitude, its effect is subordinate to the directional control by temperature. This unique thermal sensitivity is the key feature enabling the device to harness diurnal temperature cycles for sustainable operation.

To maximize power generation, we systematically optimized the device composition and geometry. In particular, incorporating Nafion proved to be crucial. As confirmed by material characterization (Figures S17 and S18), the addition of this polyanionic electrolyte significantly enhanced the electrical output, boosting the open‐circuit voltage (*V_oc_*) from 141 to 216 mV and the *I_sc_* from 0.69 µA to 1.25 µA (Figure 2d,e). This enhancement is attributed to the abundance of sulfonic acid groups in Nafion, which facilitate proton transport within the hydrogel matrix. Furthermore, geometric optimization identified a thickness of 0.8 mm and a working area of 1 cm^2^ as the optimal configuration for balancing internal resistance with moisture exchange (Figures S19 and S20). Under these optimized conditions, the device achieved a maximum power density of 16 µW cm^−2^ (volumetric power density of 200 µW cm^−3^) at a load resistance of 30 kΩ w (Figures 2f and S21 and S22). To assess stability under static conditions, we monitored the continuous discharge through a load resistor at constant temperature and humidity. The device sustained current generation for over 52 h (Figure S23). Notably, although the voltage dipped slightly from 0.5 to 0.45 V after 20 h of discharge, it spontaneously recovered to 0.5 V within 4 h, demonstrating a robust self‐charging capability that further contributes to its long‐term sustainability.

### 2.3 Temperature‑Regulated Mechanism

**FIGURE 3:**
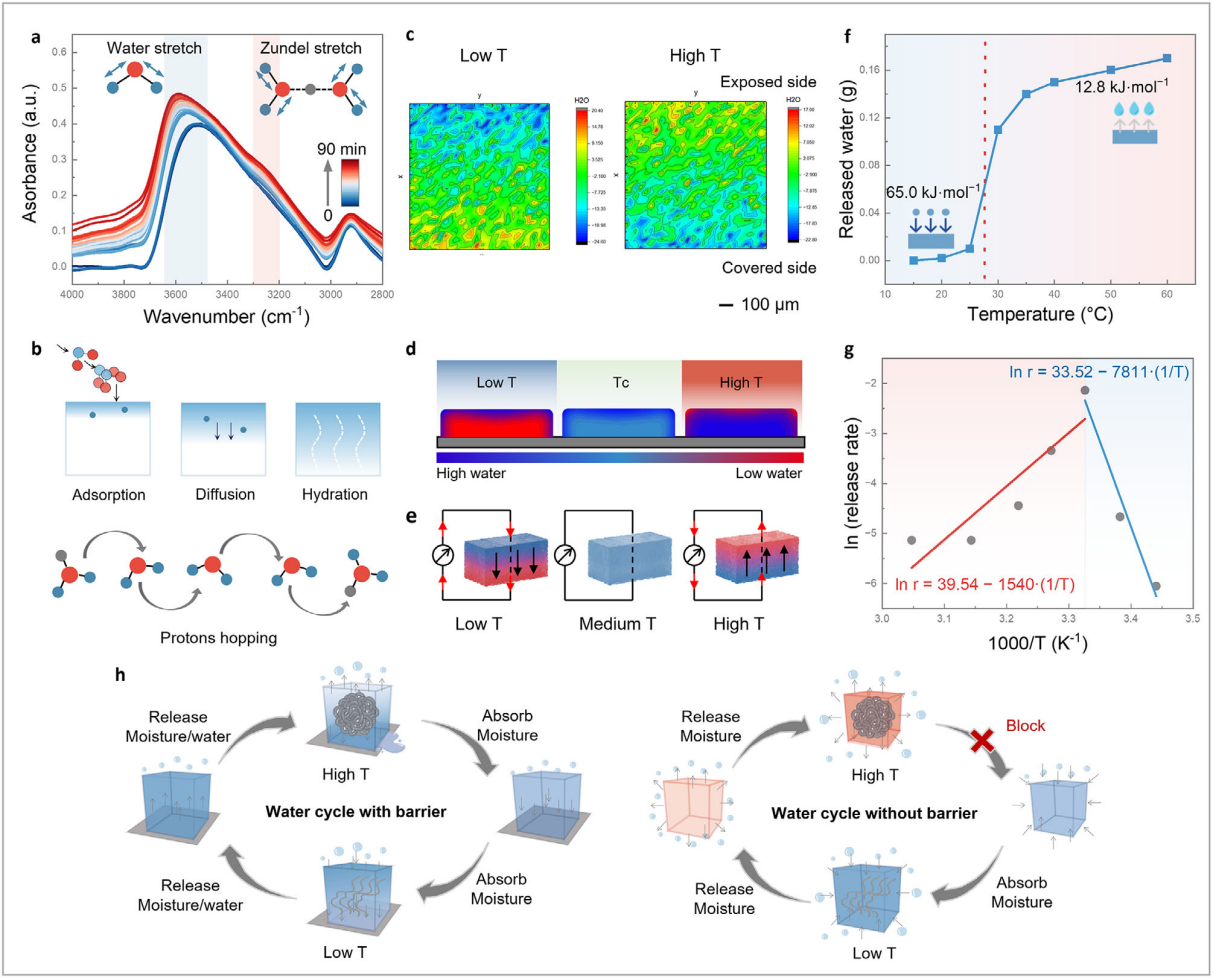
Mechanism of temperature‑regulated moisture dynamics and proton transport in the proposed hydrogel MEG. (a) In situ FTIR spectra of PVA‐SA‐Nf hydrogel exposed to ambient atmosphere (25°C, 60% RH) over 90 min. (b) Schematic illustration of the dynamic hydration process (adsorption, diffusion, hydration) and the Grotthuss proton hopping mechanism. (c) Raman mapping of water distribution on the cross sections of the MEG hydrogels. (d) Finite element simulations (COMSOL) visualizing the cross‐sectional water concentration gradients corresponding to the thermal states in (c). (e) Circuit diagrams depicting the reversible current direction driven by temperature‑induced, oscillating moisture gradients at different temperatures. (f) Gravimetric analysis of water release from the hydrogel as a function of temperature (heating rate: 1°C/min). (g) Arrhenius plots of the natural logarithm of the transition rate versus the reciprocal of the absolute temperature for water release from the hydrogel in two states. (h) Schematic comparison of the water cycle in hydrogels with and without a hindered release barrier under cyclic temperature circulation.

We first probe how Nafion and absorbed water cooperate to build fast proton‑transport pathways. To clarify the moisture absorption kinetics and the effect of the polyelectrolyte (Nafion), time‐resolved Fourier Transform Infrared (FTIR) spectroscopy was performed on dried hydrogel films exposed to ambient humidity (Figure 3a). The spectra reveal a progressive evolution in two regions over a 90‐min period. The broad absorption band centered around 3600 cm^−^^1^, characteristic of O─H stretching vibrations of water molecules, exhibits a continuous intensity increase, confirming the hydrogel's robust hygroscopicity and the substantial diffusion of water molecules into the bulk matrix. Second, a pronounced increase appears around 3200 cm^−^^1^. This might be assigned to the “Zundel stretch”, which corresponds to the vibrational mode of Zundel cations (H_5_O_2_^+^), signaling the formation of strongly hydrogen‐bonded protonated water clusters [34]. Together, these features support the three‑stage dynamic process illustrated in Figure 3b: initial surface adsorption, subsequent bulk diffusion and eventual hydration of the network. The formation of Zundel cations is driven by the dissociation of Nafion's sulfonic acid groups (─SO_3_H) in the presence of absorbed water. The resulting hydrated protons form a continuous hydrogen‑bonded network and are transported at high speed via the Grotthuss mechanism (proton hopping) (Figure 3b). The formation of Zundel‐type protonated water clusters indicates that absorbed water participates in proton conduction. These hydrogen‐bonded water clusters bridge ionic groups and support Grotthuss‐type proton hopping, thereby linking moisture uptake to enhanced electrical output.

We then resolve how temperature reshapes the internal water distribution across the asymmetric hydrogel. Building upon this molecular understanding, we investigated the macroscopic spatial distribution of water using Raman mapping on hydrogel cross‐sections under varying thermal conditions (Figure 3c), which demonstrates a reversible water gradient. At low temperatures (∼21°C), the exposed surface exhibits higher water content than the covered interface, indicating an active absorption phase. Conversely, at elevated temperatures (∼28°C), the gradient inverts, with the exposed surface becoming drier than the covered side, signifying desorption. An intermediate state (∼25°C) shows uniform water distribution, representing equilibrium. These experimental observations are quantitatively reproduced by finite element simulations (COMSOL, Figure 3d), which confirm the formation of temperature‑dependent internal water‑concentration gradients. The Raman mapping and COMSOL results correlate well with the polarity‐reversible current response. This reversible gradient inversion serves as the fundamental thermodynamic force driving directional ion migration, leading to the observed inversion of electrical output under cooling and warming cycles. As schematically illustrated in Figure 3e, the direction of the internal ionic current is set by the concentration gradient of mobile protons: at higher temperatures, ions move from the wetter covered side to the drier exposed side, while at lower temperatures the flow is reversed. By periodically inverting the water gradient, the system avoids permanent homogenization and thus circumvents the gradient exhaustion that limits conventional MEGs.

In particular, we quantify the temperature thresholds and energy barriers governing moisture uptake and release. The sustainable electricity generation of the PVA‑SA‑Nf hydrogel therefore arises from temperature‑regulated moisture uptake and release, driven by ambient thermal fluctuations. Although pure PVA is not typically thermo‐responsive, the composite hydrogel exhibits a pseudo‐phase‐transition behavior analogous to a lower critical solution temperature system [35, 36]. This behavior is attributed to temperature‑modulated physical crosslinking and hydrogen bonding, which collectively tune the effective hydrophilicity of the network [37, 38]. Gravimetric analysis reveals a distinct thermal threshold: continuous weight loss occurs above 24°C, while weight gain dominates below 26°C (Figure S24), indicating a switch from a desorption‑dominated to an absorption‑dominated regime. Kinetic analysis using Arrhenius plots (Figure 3f,g) uncovers the energy barriers governing these transitions. The desorption phase is characterized by a remarkably low activation energy (Ea) of 12.8 kJ mol^−^^1^, suggesting a facile process likely involving the seepage of liquid‐like water clusters rather than pure vaporization. In contrast, the absorption phase presents a significantly higher Ea of 65.0 kJ mol^−^^1^, consistent with the chemisorption of gaseous moisture, which requires overcoming a larger energy barrier for network reconfiguration. Taken together, these results reveal that Nafion‑enabled proton networks and temperature‑driven reversible water‑gradient inversion jointly sustain long‑term moisture‑powered electricity generation.

Crucially, the nanoporous polyethylene (PE) membrane acts as a diffusive barrier that induces a critical “hindered release” effect (Figure 3h). In an unhindered configuration, unrestricted evaporation during high‐temperature phases may lead to severe dehydration and structural collapse, thereby blocking subsequent re‐absorption. By contrast, the PE membrane imposes a kinetic restriction on water loss. This design retains a fraction of bound water even during desorption, functioning as a built‐in water reservoir. This mechanism prevents the hydrogel from reaching a completely desiccated state, thereby preserving the ionic conductivity essential for continuous operation. To further distinguish the self‐sustaining output from a transient humidity‐response effect, we measured the electrical output under constant humidity conditions at 25°C and 30%–70% RH (Figure S25). The current gradually decayed under fixed RH, indicating that a static humidity environment cannot continuously regenerate the internal water gradient. In contrast, diurnal temperature/humidity variations can dynamically reverse and rebuild the water gradient, enabling cyclic self‐sustaining output. Consequently, under diurnal temperature cycling, the hydrogel maintains a dynamic non‐equilibrium state, oscillating between regulated moisture loss and re‐absorption. This thermodynamic regulation ensures long‐term sustainability and produces the characteristic alternating current output that mirrors the reversible inversion of the internal moisture gradient.

The enhanced MEG output originates from the coupling of material structure, moisture redistribution, and hydrated ion transport. The PVA‐SA‐CaCl_2_ network provides moisture adsorption and water retention, Nafion supplies sulfonic‐acid‐rich pathways for proton transport, and the nanoporous PE layer establishes asymmetric moisture exchange. Under diurnal temperature fluctuations, reversible adsorption/desorption generates alternating water gradients across the hydrogel, which drive directional ion migration and lead to polarity‐reversible electrical output. This dynamic non‐equilibrium mechanism prevents saturation or desiccation‐induced performance decay and enables sustained MEG operation.

### 2.4 Preliminary Extension of the Diurnal Moisture‐Electric Concept

**FIGURE 4:**
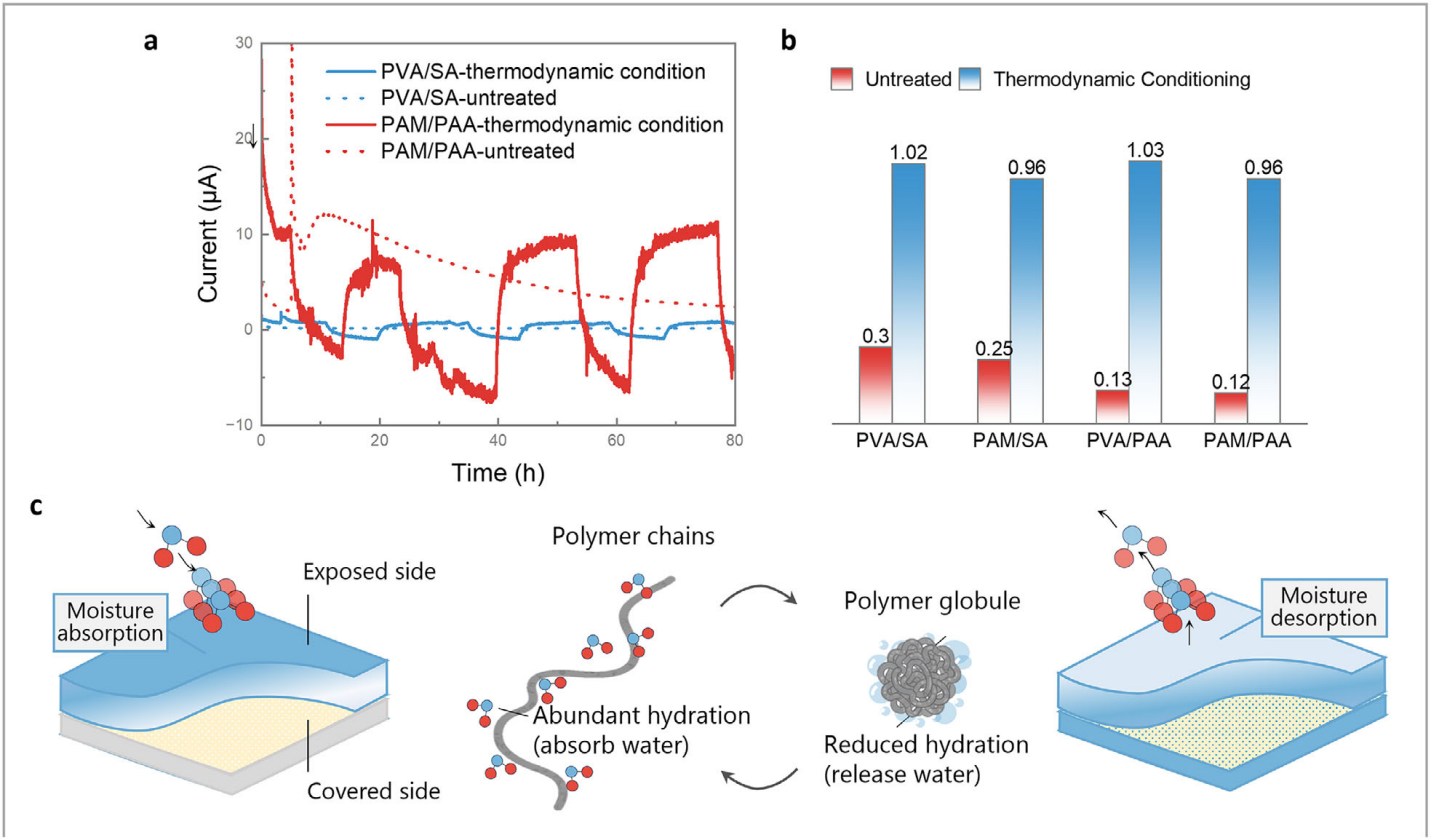
Transferability of the temperature‑driven strategy and preliminary extension of the design principle. (a) Comparative temporal current profiles of MEGs based on PVA/SA (blue) and PAM/PAA (red) hydrogels. Dashed curves denote devices operated under static conditions (no thermal cycling), whereas solid curves correspond to devices subjected to cyclic thermodynamic conditioning. (b) Comparison of current retention from the maximum current output on Day 3 relative to Day 1 for PVA, PAM, PAA, and SA hydrogels under static‐treated conditions versus thermodynamic treatment conditions. (c) Generalized design roadmap for temperature‑induced, long‑lived MEGs, integrating hierarchical considerations from molecular‑level functional group selection and ionic carriers, through structural network engineering for water retention and stability, to asymmetric device‑interface design and the critical dynamic thermodynamic operating protocol.

To examine whether the reversible moisture‐electric behavior observed in the PVA‐SA‐Nf system is restricted to a single formulation, we further evaluated several related hydrophilic polymer systems, including combinations based on PVA, polyacrylic acid sodium salt (PAA), and polyacrylamide (PAM). As illustrated in Figure 4a, under static conditions without cyclic conditioning, devices based on PVA/SA and PAM/PAA exhibit much weaker attenuated current output over time. By contrast, when subjected to cyclic thermodynamic conditioning, these related systems display oscillatory electrical responses over the 80 h testing period, indicating that the reversible output behavior is not limited to one exact hydrogel composition.

The superiority of this strategy is further evaluated by the current retention ratio. As summarized in Figure 4b, devices in the static control group retain less than 25% of their initial performance, reflecting the gradual approach to a near‑equilibrium state and the consequent dissipation of the driving moisture gradient. Conversely, the thermodynamically conditioned group maintains exceptional retention rates ranging from 95% to 103%. This result suggests that cyclic thermal treatment can help sustain reversible moisture‐electric behavior in the hydrogel systems examined here, likely by promoting repeated sorption/desorption‐associated water redistribution rather than allowing the device to evolve monotonically toward a persistent saturated or dried state. This active modulation ensures a continuous renewal of the moisture gradient, preventing the dissipation of the driving force for ion transport.

Based on these findings, we summarize several provisional design considerations that appear to be relevant for achieving this type of reversible behavior in the presently examined systems (Figure 4c). At the molecular level, polymers with abundant hydrophilic groups (e.g., ─OH, ─COOH) and mobile ions are selected to ensure high hygroscopicity and ionic conductivity. At the structural level, a crosslinked network that balances water retention with mechanical stability should be constructed. At the device level, an asymmetric structure with exposed (solid‐gas) and covered (solid‐solid) interfaces to facilitate a water gradient is necessary. At the operational level, alternating high‑ and low‑temperature treatments induce reversible polymer‑chain dynamics between expanded and contracted states, thereby constructing and periodically inverting internal water gradients, preventing saturation or complete drying, and driving continuous energy harvesting. These features suggests as preliminary design insights for related hydrogel MEGs with sustainable generation.

### 2.5 Scalability and Applications

**FIGURE 5:**
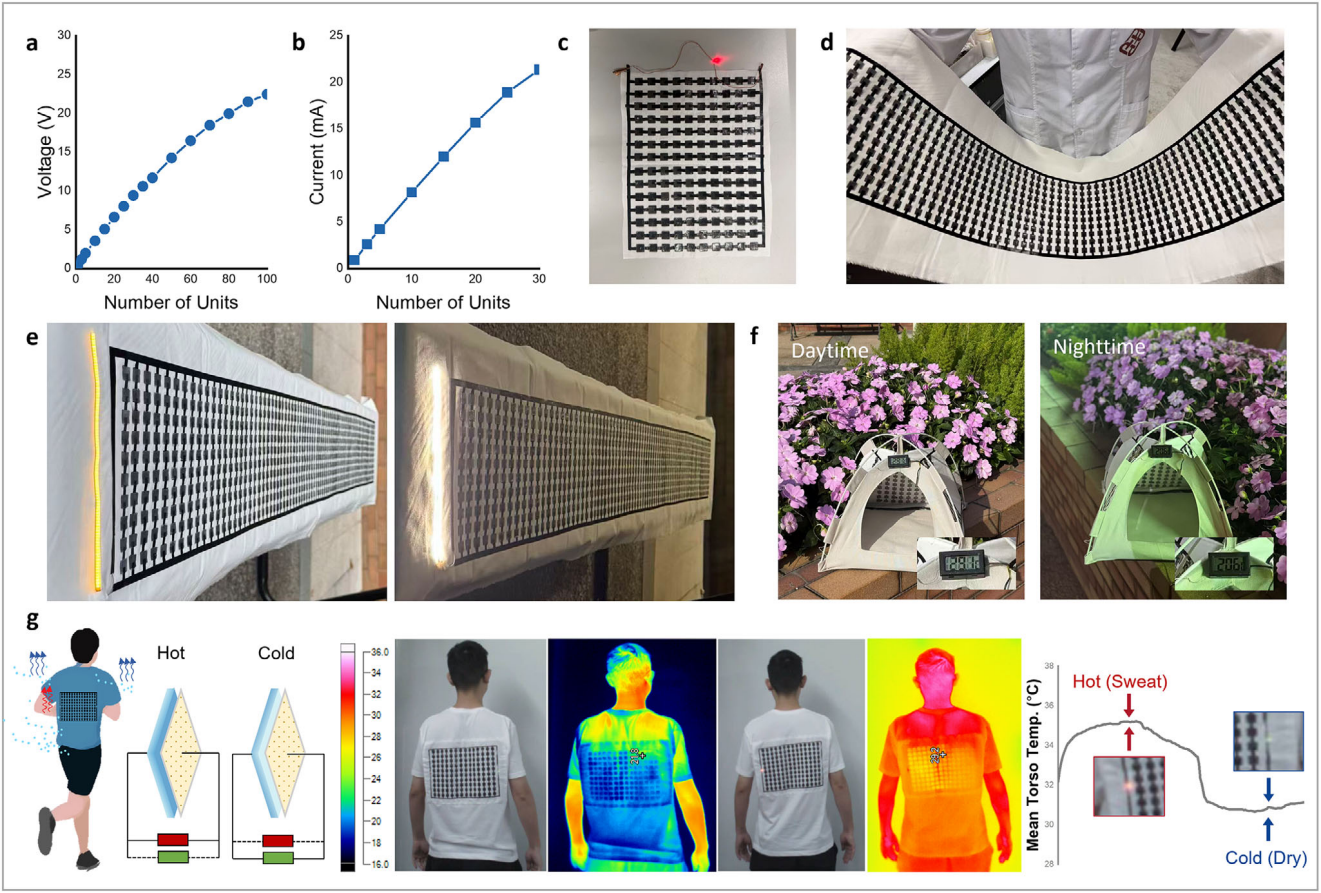
System integration, scalability, and on‑body evaluation of the temperature‑driven MEG system. Electrical output performance of the MEG units, showing the (a) *V_oc_* and (b) *I_sc_* scaling linearly with the number of units connected in series and parallel, respectively. (c) Photograph of a textile module consisting of 120 MEGs (1.5 cm × 1.5 cm × each), configured as 12 parallel branches with 10 MEGs connected in series in each branch, used to directly power a red LED and demonstrate basic functionality. (d) Photographs demonstrating the large‐scale integration and flexibility of a foldable 800‐unit 1.5 cm ×1.5 cm MEG sheet, 80 parallel branches with 10 MEGs connected in series. (e) Demonstration of the MEG sheet driving a commercial LED strip outdoors. (f) Circuit diagram and demonstration of a smart tent powering a temperature sensor integrated with 210 1.5 cm ×1.5 cm × 1.5 mm MEG units, 21 parallel branches with 10 MEGs connected in series. All MEGs The photographs capture the setup during daytime (29.1°C) and nighttime (20.6°C) environments. (g) Conceptual illustration, circuit design, optical photographs, and infrared images of a subject wearing the MEG‐integrated T‐shirt. The circuit is designed to function as a self‐powered temperature indicator: the green LED activates under low‐temperature conditions (subject feels cold), while the red LED illuminates under high‐temperature conditions (subject feels hot). Detailed information of the demonstrations is summarized in Table S2.

To bridge the gap between laboratory prototypes and practical power sources, we evaluated the scalability and integration potential of the MEGs. We first constructed sequentially aligned MEG arrays using highly conductive paste to minimize contact resistance at the hydrogel‐electrode interface, ensuring efficient charge transfer. Systematic testing under constant environmental conditions reveals that both open‐circuit voltage (*V_oc_*) and short‐circuit current (*I_sc_*) scale linearly with the number of units connected in series and parallel, respectively (Figure 5a,b). This linear scalability confirms that the internal impedance of individual units is consistent and sufficiently low to support large‐scale integration without significant power loss. As a proof of concept, these integrated arrays successfully powered commercial electronics, such as LEDs (Figure 5c). To further demonstrate macroscopic energy harvesting, we fabricated a large‑scale, flexible MEG sheet on a bendable substrate (Figure 5d,e). This flexible and foldable system generated sufficient power to drive a commercial LED strip.

Beyond simple power generation, we leveraged the unique temperature‐dependent current reversal characteristic of the MEG to design a smart, self‐powered sensing system. This built‑in energy‐sensing duality allows the device to act as an active environmental monitor. We integrated 210 MEG units onto flexible fabrics to assemble a smart tent using a hybrid series‐parallel connection (Figure 5f). To visualize the environmental thermal state, the circuit was configured to power a digital indicator. During the daytime (high temperature, ∼29.1°C), the system generated a specific polarity current to activate the monitor; crucially, during the nighttime (low temperature, ∼20.6°C), the current direction reversed but continued to power the device via a bi‐ bidirectional circuit design, thereby validating the continuous energy harvesting capability driven by ambient temperature fluctuations.

Furthermore, we extended the design concept to bio‐integrated electronics by fabricating a personal thermal management T‐shirt equipped with an expanded polytetrafluoroethylene (ePTFE) membrane as a moisture modulation barrier (Figure S26). The working mechanism relies on the moisture gradient established by the temperature contrast between the human skin and the external environment. As illustrated in Figure 5g, when the wearer experiences heat (high body temperature/perspiration), the hydrogel on the skin‐contact side undergoes thermally induced desorption and establishes a pronounced moisture gradient, driving a current direction that illuminates a red LED (“Hot” Signal). Conversely, when the wearer feels cold (low ambient temperature), the skin side remains relatively cooler, and the gradient inverts due to the environmental thermal shift, reversing the current flow to light up a green LED (“Cold” Signal). This application underscores the versatility of the thermodynamic‐conditioned MEG: it functions not only as a sustainable energy source but also as a self‐powered, logic‐enabled sensor. This integration of energy harvesting and physiological monitoring holds great promise for the next generation of smart textiles and autonomous wearable health systems.

## 3 Conclusions

In summary, we have developed a highly sustainable and thermodynamically modulated MEG based on asymmetric PVA‐SA‐Nf hydrogel inspired by circadian rhythm. By fundamentally reimagining the role of environmental fluctuations, we transform ambient temperature variations—traditionally considered detrimental noise—into a critical driving force that regulates the hydrogel's hydration state. This thermodynamic conditioning strategy effectively overcomes the structure damage bottleneck inherent in conventional hydrogel‐based MEGs, enabling the device to maintain a dynamic non‐equilibrium state characterized by reversible current direction and water gradient. Our mechanistic investigation reveals that the system operates through a temperature‐regulated moisture exchange process. The discovery of a distinct transition temperature for water absorption and desorption drives a reversible water concentration gradient across the hydrogel, which serves as the fundamental engine for ionic transport. Coupled with the hindered release effect that prevents desiccation, this unique interplay between polymer physics and structural engineering endows the device with exceptional durability, demonstrating continuous, self‐recovering electricity generation for over 57 days under natural thermal cycling. Beyond superior stability, we demonstrated the MEG's scalability and smart integrated applications. The integrated MEG arrays not only provide reliable power for commercial electronics but also enable intelligent sensing capabilities. Leveraging the intrinsic current reversal characteristic, we prototyped a self‐powered smart T‐shirt for personal thermal management capable of visually indicating human thermal sensation without an external circuit. This work provides an improved understanding of hydrogel thermodynamics and offers a transferable design paradigm for developing maintenance‑free energy harvesters and self‑aware sensors for next‑generation intelligent wearable ecosystems. Nevertheless, fully exploiting the mechanical robustness and fatigue resistance of the hydrogel network, particularly under harsh environments and repeated thermal cycling, will unlock additional performance beyond what is shown here. Future studies will integrate tougher polymer architectures and reinforcing strategies to further strengthen mechanical durability while maintaining the desirable thermodynamic response.

## 4 Experimental Section

### 4.1 Materials

PVA (87%–89% hydrolyzed, high molecular weight), PAM (Mw ∼ 5000,000), PAA (30 wt.%, Mw ∼5,000), SA (low viscosity), CaCl_2_ (anhydrous, porous, 93%) were purchased from Thermo Scientific (Alfa Aesar). Nafion 117 (∼5 wt.% in a mixed solvent of lower aliphatic alcohols and water) was purchased from Macklin. Porous polyethylene (PE) with 25‐micron thickness was purchased from Celgard Co. Ltd.

### 4.2 Preparation of the PVA‐SA‐Nf Hydrogels

A total of 15 g PVA and 1.5 g SA were dissolved into 70 mL deionized water and stirred at 90 C for 12 h. Then, 10 mL of 30 wt.% CaCl_2_ solution was added to the mixture. The solution was divided into five equal portions; Nafion solution (100, 200, 300, and 400 µL, respectively) was added to four of the portions, and all samples were stirred at room temperature for 24 h. Finally, the mixtures were cast into five Petri dishes (diameter 8.5 cm), each containing ∼15 g of precursor solution. For comparison, 2 g PAM and 5 g PAA were dissolved in 67 mL deionized water and stirred at 60°C for 12 h to ensure complete dissolution and mixing. The subsequent addition of CaCl_2_ and Nafion, as well as the post‑treatment procedures, followed the same protocol as for the PVA‑SA‑Nf hydrogels.

### 4.3 Preparation of MEGs

Au electrode is thermally evaporated (Denton Thermal Evaporator) onto a porous PE film with a thickness of 70 nm at a rate of 0.5 Å/s. The PVA‐SA‐Nf hydrogel is adhered onto the Au side, and graphene is dry‐coated onto the other side of the hydrogel. Copper‐conducted electrodes are connected with the MEG and Keithley 2400 to test the electrical performance. The as‑prepared PVA‑SA‑Nf hydrogel MEGs were placed under indoor air‑conditioning (22°C–28°C) for three days (as thermodynamic condition). For comparison, control hydrogels were thermally treated in an oven at 60°C for 2 h (as oven‐conditioned) and in a chamber at RH 100% (as humidity‐conditioned)

### 4.4 Characterization, Measurement, and Simulation

SEM images and Energy Dispersive X‐Ray Spectroscopy (EDX) were all taken by Tescan VEGA. Electrical properties were tested by Keithley 2400. In situ FTIR was tested by Spectrum 100, PerkinElmer. Raman mapping was performed under a 532 nm laser with a Renishaw Micro–Raman Spectroscopy System with a dimension of 0.25 mm × 0.25 mm. Raman intensity was selected at 3280 cm^−1^ which corresponds to the symmetric O─H stretching mode in H_2_O. All weights were measured by a balance with a precision of 0.01 mg. Subject skin temperatures are measured with ibuttons DS1923‐F5, and mean skin temperature is calculated by the equation: ${\bar{T}}_{torso}=0.25\left(T_{chest}+T_{scapula}+T_{abdomen}+T_{lumbar}\right)$. Water content of the hydrogel was simulated using COMSOL Multiphysics. In this model, the liquid and gaseous phases are assumed to be in equilibrium inside the hydrogel sample, which is treated as a porous medium. The model includes full boundary conditions: both side inlets have zero flow velocity to simulate natural convection; the top boundary is set as a pressure outlet; the bottom boundary is adiabatic with no mass flow. The inlet and outlet temperatures are both fixed at ambient temperature (293.15 K). Ambient relative humidity is 0.1, while the initial relative humidity inside the hydrogel is 0.895. Key material parameters are: porosity = 0.8, permeability = 1 × 10^−^^14^ m^2^. A fitting procedure was applied to calibrate any remaining unknown parameters against measured data. The model computes the transient change in water saturation to simulate coupled heat and moisture transport via two‐phase flow. Finally, the simulated results were validated against independent measurements of water‑content profiles to ensure predictive accuracy.

### 4.5 Calculation of Activation Energy

Hydrogels were placed in a lab‐made climate chamber (temperature 10°C to 30°C; RH 40%–90%). The mass m was recorded, and the unit‐mass weight change was defined as r = |Δm|. Arrhenius plot was constructed by performing a linear regression of ln r versus 1/T. The regression equation ln r = a + b·(1/T) was fitted, where b is used to calculate the apparent activation energy Ea via Ea = −b·R, with R denoting the gas constant (R = 8.314 J·mol^−1^·K^−1^). To avoid deviations from linearity arising from phase transitions or changes in the dominant transport mechanism, adsorption and desorption processes were fitted separately.

## Author Contributions

Z.L. and D.S. conceived the project. Z.L. designed and performed the experiments and prepared the figures. D.A., M.P., X.Z. and P.Z. contributed to materials preparation and device characterization. H.Y. performed the simulations. J.W. contributed to methodology. D.S. supervised the project and acquired funding. All authors discussed the results and contributed to the writing and revision of the manuscript, and approved the final version.

## Conflicts of Interest

The authors declare no conflicts of interest.

## Supporting information




**Supporting File**: advs77689‐sup‐0001‐SuppMat.docx.

## Data Availability

The data that support the findings of this study are available on request from the corresponding author. The data are not publicly available due to privacy or ethical restrictions.
