# Supplementary material for: Circadian‐Inspired Hydrogel Moisture Electricity Generation
Source: Adv Sci (Weinh). 2026 Sep 9:e77689. Online ahead of print. doi: 10.1002/advs.77689 (PMC13558901; doi:10.1002/advs.77689)
Supplement: Supplementary file 1 [file ADVS-9999-e77689-s001.docx]

**Supporting Information**

**Circadian‑Inspired Hydrogel Moisture Electricity Generation**

Ziqi Li^abc^, Dilalanmu Ahemaitijiang^a^, Hongfei Yang^a^, Xuan Zhao^a^, Mengjiao Pan^a^, Jun Wan^a^, Pingan Zhu^d^, Dahua Shou*^abc^

^a^*Future Intelligent Wear Centre, School of Fashion and Textiles, The Hong Kong Polytechnic University, Hung Hom, Kowloon, Hong Kong Special Administrative Region of China.*

*^b^Research Centre of Textiles for Future Fashion, The Hong Kong Polytechnic University, Hung Hom, Kowloon, Hong Kong Special Administrative Region of China.*

*^c^Research Institute for Intelligent Wearable Systems, The Hong Kong Polytechnic University, Hung Hom, Kowloon, Hong Kong Special Administrative Region of China.*

*^d^Department of Mechanical Engineering, City University of Hong Kong, Kowloon, Hong Kong Special Administrative Region of China.*

*Corresponding author. Email: [dahua.shou@polyu.edu.hk](mailto:dahua.shou@polyu.edu.hk)

**Figure S1**. Global distribution of the mean daily accumulated evaporation (mm day⁻¹) during 1991–2020, highlighting the ubiquity of atmospheric moisture generated by liquid/solid phase transitions and terrestrial/aquatic transpiration. Data were obtained from the Climate Data Store (CDS).

**Figure S2**. Thermodynamic conditioning protocol for the hydrogel. The PVA‑SA‑Nf hydrogel was placed under air‑conditioned indoor conditions (RH ~60%) and subjected to cyclic room‑temperature variations between 22 °C and 28 °C for 12 h before assembling and testing.

**Figure S3**. Effect of different conditioning treatments on the electrical output of MEGs. The thermodynamically conditioned device exhibits a stronger and more stable current output than the other two groups, together with the highest first-day current amplitude. These results indicate that the conditioning protocol has a pronounced effect on the subsequent reversible electrical behavior of the hydrogel MEG.

**Figure S4.** Electrical output of the MEGs with symmetric and asymmetric electrodes. To assess the possible contribution of electrode asymmetry, devices with symmetric electrodes were tested under the same conditions (25 ℃, 60% RH). The output behavior remained dependent on the hydrogel structure and environmental cycling, indicating that the reversible signal cannot be solely attributed to asymmetric electrode configuration.

**Figure S5.** Electrical response of MEGs under reversed electrode connection. Reversing the external electrode connection led to the expected change in measured signal polarity, confirming the electrical readout consistency and helping exclude trivial wiring artifacts.

**Figure S6.** Electrical output and the first-day current amplitude of MEG devices with and without CaCl₂. Results showed markedly weakened electrical output, supporting the important role of the hygroscopic salt in moisture uptake and the associated reversible electrical response.

**Figure S7.** Electrical output of MEGs under PE coverage and exposure configurations. Altering the PE coverage/exposure geometry significantly affected the output magnitude and reversibility, indicating that asymmetric moisture exchange is a key structural factor in establishing the reversible current behavior.

**Figure S8.** Electrical output of open and sealed MEGs. When external moisture exchange was suppressed by sealing, the output was significantly weakened and the reversible behavior became much less evident, supporting the importance of moisture transport and internal water redistribution in the device operation.

**Figure S9**. Benchmarking of operational stability for MEGs. Current retention as a function of operation duration for this work and reported MEGs. Current retention was estimated from the reported long-term output profiles using the ratio between the maximum current output at the final and initial stages.

**Figure S10**. Long-term self-sustaining electrical output from 31^st^ to 56^th^ day. Daily maximum and minimum short-circuit currents were extracted from the continuous output profile using a 24 h interval. The current difference, defined as $\Delta I=I_{max}-I_{min}$, was calculated to quantify the daily output amplitude. The MEG maintained measurable daily current fluctuations over ~57 days, confirming its long-term self-sustaining operation capability.

**Figure S11.** Current output characteristics of the MEG as a function of temperature under different ambient relative humidities (50%–66%).

**Figure S12**. Electrical output og PVA-SA-Nf MEG under fixed relative humidity of 60% and varying temperatures. At a constant relative humidity of 60%, the device output still changed systematically with temperature. This result indicates that thermal variation affects the electrical response even when RH is held constant, suggesting that the device behavior cannot be explained solely by a simple monotonic dependence on RH.

**Figure S13.** Current output as a function of temperature at an RH of 52%.

**Figure S14.** Current output characteristics of the MEG as a function of humidity at different ambient temperatures (17–29 °C).

**Figure S15**. Electrical output under fixed temperature and varying relative humidity. At a fixed temperature of 23 °C, changing the relative humidity also altered the electrical output. This confirms that moisture conditions remain an important contributing factor and further supports that the device behavior is governed by coupled thermal–moisture conditions rather than by temperature alone.

**Figure S16.** Current output at 21°C and 28°C as a function of relative humidity.

**Figure S17.** SEM images of the freeze‑dried PVA‑SA‑Nf hydrogel and EDS elemental mapping of C, O, Cl, Ca, and F. The results confirm a uniform spatial distribution of all elements within the hydrogel network.

**Figure S18**. Impact of Nafion content on the electrical performance of PVA-SA-Nf hydrogel-based MEGs.

**Figure S19.** Impact of hydrogel thickness on the open‑circuit voltage (Voc) and short‑circuit current (Isc) of PVA‑SA‑Nf MEGs.

**Figure S20.** Influence of the effective working area on the open‑circuit voltage (Voc) and short‑circuit current (Isc) of PVA‑SA‑Nf MEGs.

**Figure S21.** Representative output power density of the device as a function of external load resistance, showing that an external resistance of 30 kΩ yields the optimal output performance. A total of 12 independently fabricated devices were tested. Among them, 11 devices were functional and 1 device failed, corresponding to a fabrication yield of 91.7%. For the valid devices, the power output was 95.6 ± 1.0 μW for a device area of 2 cm × 2 cm, corresponding to a power density of 23.9 ± 0.25 μW cm^−2^.

**Figure S22.** Short-term output retention of three independently fabricated MEG devices. Electrical output of three independently fabricated MEG devices measured over a shorter monitoring period under the same test condition. The results show consistent retention behavior across replicate samples, providing additional support for the reproducibility of the reversible output under the tested conditions.

**Figure S23.** Evolution of I (red) and V (black) of MEG in a constant temperature and humidity environment (RH 56%, T 23°C). The initial V of the device is about 0.48 V (from 0 to 4 hours). Connecting to the load resistance (RL = 30 kΩ) produces a continuous and gradually stable I of about 680 nA for 20 hours (red curve, 4 hours to 24 hours). Then the RL is disconnected, and V is recorded again, and V gradually increases to the initial value of 0.51V (from 24 h to 28 h), showing a self-charging process. Reconnect to the R_L_, which produces repeated continuous power supply (I about 666nA) (red curve, 28 - 48 hours). Disconnecting the R_L_ produces a second self-charging process (48 - 52 hours), bringing the V back to 0.5V again. The diagram on the left shows the test circuit diagram.

**Figure S24**. Mass change of the hydrogel as a function of time under different temperature conditions.

**Figure S25**. Electrical output of the constant-humidity electrical-output measurement. The hydrogel MEG was placed in a temperature–humidity chamber and tested at 25 °C under 30%, 40%, 50%, 60%, and 70% RH. The 20-hours constant-humidity measurements demonstrate continuous, humidity-dependent electrical output, with a polarity transition from low to high RH, supporting that the MEG behavior is governed by humidity-regulated water redistribution and directional ion transport rather than a simple transient humidity-change response.

**Figure S26**. Schematic illustration of the fabrication process of the wearable MEG T‑shirt, including ePTFE hot‑pressing, screen printing of electrodes, hydrogel attachment, and connection with conductive tapes.

**Figure S27.** Electrical output of the textile module consisted of 120 MEGs. The textile module exhibited an initial $I_{sc}$of approximately 8 mA, followed by a gradual decrease and stabilization at ~2.6–2.8 mA over 10,000 s. The corresponding $V_{oc}$remained in the volt-level range throughout the measurement. These results provide quantitative evidence for the sustained electrical output of the MEG-based textile module.

**Table S1**. Benchmarking of operational stability for MEGs.

| Ref. | Operation duration / days | First-day current / μA | Last-day current / μA | Current retention / % |
| --- | --- | --- | --- | --- |
| (*1*) | ~26 | ~2.2 | ~0.45 | ~20 |
| (*2*) | 10 | ~8.0 | ~7.1 | ~89 |
| (*3*) | ~0.49 | ~0.015 | ~0.0055 | ~37 |
| (*4*) | ~0.67 | ~300 | ~110 | ~37 |
| (*5*) | ~33 | ~1.45 | ~0.10 | ~7 |
| (*6*) | 10 | ~8.7 | ~7.1 | ~82 |
| (*7*) | 5 | ~3.6 | ~2.5 | ~69 |
| This work | ~57 | ~0.80 | ~0.90 | ~89 |

Note: The first-day and last-day current values were estimated from the reported long-term output profiles. For devices with cyclic or fluctuating outputs, the maximum peak current values within the first and last day were used for calculating current retention.

**Table S2**. Performance metrics of MEG-powered application demonstrations.

| Demonstration | MEG units | Voc/V | Isc/mA | P/mW | Operation mode |
| --- | --- | --- | --- | --- | --- |
| Textile module | 120 | 1.8 | 2.5 | 4.5 | Direct |
| Large-area sheet | 800 | 6.5 | 20 | 130 | Capacitor-assisted |
| Smart tent | 210 | 2.0 | 3.8 | 7.5 | Capacitor-assisted |
| Smart T-shirt | 140 | 2.5 | 2.1 | 5.1 | Direct |

Note: The operating voltage and current refer to the values measured during practical load operation. The device power was calculated using $P=VI$ or obtained from the measured voltage. For capacitor-assisted demonstrations, the operation power corresponds to the discharge stage powered by the stored electrical energy.

References

1.	P. Duan *et al.*, Moisture-based green energy harvesting over 600 hours via photocatalysis-enhanced hydrovoltaic effect. *Nat Commun* **16**, 239 (2025).

2.	J. Tan *et al.*, Self-sustained electricity generator driven by the compatible integration of ambient moisture adsorption and evaporation. *Nat Commun* **13**, 3643 (2022).

3.	K. Zhao *et al.*, Moisture-driven energy generation by vertically structured polymer aerogel on water-collecting gel. *Nano Energy* **126**, (2024).

4.	S. Yang *et al.*, Green moisture-electric generator based on supramolecular hydrogel with tens of milliamp electricity toward practical applications. *Nat Commun* **15**, 3329 (2024).

5.	Y. Zhang *et al.*, Sustainable power generation for at least one month from ambient humidity using unique nanofluidic diode. *Nat Commun* **13**, 3484 (2022).

6.	X. Liu *et al.*, Power generation from ambient humidity using protein nanowires. *Nature* **578**, 550-554 (2020).

7.	Z. Sun, X. Wen, L. Wang, J. Yu, X. Qin, Capacitor-inspired high-performance and durable moist-electric generator. *Energy & Environmental Science* **15**, 4584-4591 (2022).
